# Combination of blood oxygen level–dependent functional magnetic resonance imaging and visual evoked potential recordings for abnormal visual cortex in two types of amblyopia

**Published:** 2012-04-11

**Authors:** Xinmei Wang, Dongmei Cui, Ling Zheng, Xiao Yang, Hui Yang, Junwen Zeng

**Affiliations:** 1 State Key Laboratory of Ophthalmology, Zhongshan Ophthalmic Center, SunYat-sen University, Guangzhou, P.R. China; 2 Department of Ophthalmology, The Fourth Affiliated Hospital, Harbin Medical University, Harbin, P.R. China

## Abstract

**Purpose:**

To elucidate the different neuromechanisms of subjects with strabismic and anisometropic amblyopia compared with normal vision subjects using blood oxygen level–dependent functional magnetic resonance imaging (BOLD-fMRI) and pattern-reversal visual evoked potential (PR-VEP).

**Methods:**

Fifty-three subjects, age range seven to 12 years, diagnosed with strabismic amblyopia (17 cases), anisometropic amblyopia (20 cases), and normal vision (16 cases), were examined using the BOLD-fMRI and PR-VEP of UTAS-E3000 techniques. Cortical activation by binocular viewing of reversal checkerboard patterns was examined in terms of the calcarine region of interest (ROI)-based and spatial frequency–dependent analysis. The correlation of cortical activation in fMRI and the P_100_ amplitude in VEP were analyzed using the SPSS 12.0 software package.

**Results:**

In the BOLD-fMRI procedure, reduced areas and decreased activation levels were found in Brodmann area (BA) 17 and other extrastriate areas in subjects with amblyopia compared with the normal vision group. In general, the reduced areas mainly resided in the striate visual cortex in subjects with anisometropic amblyopia. In subjects with strabismic amblyopia, a more significant cortical impairment was found in bilateral BA 18 and BA 19 than that in subjects with anisometropic amblyopia. The activation by high-spatial-frequency stimuli was reduced in bilateral BA 18 and 19 as well as BA 17 in subjects with anisometropic amblyopia, whereas the activation was mainly reduced in BA 18 and BA 19 in subjects with strabismic amblyopia. These findings were further confirmed by the ROI-based analysis of BA 17. During spatial frequency–dependent VEP detection, subjects with anisometropic amblyopia had reduced sensitivity for high spatial frequency compared to subjects with strabismic amblyopia. The cortical activation in fMRI with the calcarine ROI-based analysis of BA 17 was significantly correlated with the P_100_ amplitude in VEP recording.

**Conclusions:**

This study suggested that different types of amblyopia had different cortical responses and combinations of spatial frequency–dependent BOLD-fMRI with PR-VEP could differentiate among various kinds of amblyopia according to the different cortical responses. This study can supply new methods for amblyopia neurology study.

## Introduction

Amblyopia, one of the leading causes of vision loss in children, affects approximately 1.6%–3.6% of the general population [1–4]. Early visual experience is abnormal under such conditions as strabismus, in which the visual axes are misaligned, and anisometropia, in which the refractive state is significantly different between the two eyes. The two conditions often lead to monocular amblyopia without a macroscopic lesion along the visual pathway. The pathogenetic mechanism of amblyopia may differ in the two conditions [5], since strabismus prevents concordant stimulation of binocularly corresponding points in the two retinas, whereas anisometropia imposes constant blurring of one retinal image relative to the other. Clinical studies on human patients with amblyopia confirmed that the two conditions lead to different forms of amblyopia [6,7]. Although this issue remains controversial [8,9], experiments using several animal models have suggested that strabismic amblyopia results from the breakdown of binocular interaction [10–16]. In contrast, reduction in the spatial resolution of neurons with preferred spatial frequency in the higher range or the loss of such neurons may underlie anisometropic amblyopia [6,17,18].

Neurophysiological studies have provided a great deal of evidence regarding the functional effects of vision deprivation in the visual cortex [14,15,19–21]. The primary visual cortex was considered the principal site of vision deficit, whereas studies have also shown the extrastriate cortex is responsible for abnormalities of special visual function [22–24]. However, neuroanatomic investigations of these abnormalities remain uncertain. Only recently has cortical activity in human amblyopes been studied [21,25–28]. Blood oxygen level–dependent functional magnetic resonance imaging (BOLD-fMRI) uses the focal uncoupling of cerebral blood flow and metabolism to detect focal brain activation [29–34]. fMRI is considered to have an accurate spatial resolution, as low as 0.9 mm [35], which has allowed researchers to study with some precision the spatial organization of the brain within and across visual areas. However, this technique has limited ability to reveal the temporal dynamics of cortical areas. The ideal brain-imaging system provides high spatial and temporal resolution. fMRI and pattern-reversal visual evoked potential (PR-VEP) detect two fundamentally different physiologic phenomena reflecting brain activity: fMRI typically measures changes in blood oxygenation due to neuronal activity, whereas VEP measures the electric potential directly generated by neuronal activity. The fMRI (hemodynamic) and VEP (electric) measurements are complementary in their spatiotemporal resolutions. fMRI has high spatial resolution, typically on the order of millimeters, whereas VEP has millisecond temporal resolution. Because these methods are sensitive to different temporal and spatial properties of brain function, they have the potential to complement one another. The low time resolution of BOLD sampling blurs interpretations of the functional specificity of a BOLD-activated brain structure. Peaks and latencies of VEP provide additional functional information at a high time resolution. They can be used to describe functional fMRI-activations more precisely [36–42].

Although some studies have been performed on simultaneous VEP/fMRI recording in neuroscience, this area is still in the early stages. Moreover, no work has combined these two methods to explore neuro-ophthalmology problems. The aim of the present study was to examine the cortical response and the deficit site of strabismic and anisometropic amblyopia with spatial frequency–dependent BOLD-fMRI and PR-VEP. In addition, this study sought to evaluate the differences in the response pattern between the two types of amblyopia and determine whether the cortical activation of fMRI is correlated with the VEP recording.

## Methods

### Subjects

Fifty-three subjects (twenty-eight male and twenty-five female, mean age 8.82±1.47 years, ranged from 7 to 12 years old) who 20 patients with monocular anisometropic amblyopia, 17 patients with monocular strabismic amblyopia, and 16 normal vision volunteers participated in this study. All subjects had no history of physical disorders. None of the subjects had a history of other ophthalmological or systemic diseases and were diagnosed by experts at the Zhongshan Ophthalmic Center between November 2004 and March 2006. All subjects completed an ophthalmologic examination to confirm the diagnosis; this included ocular motility tests, dilation, a fundus exam, autorefraction, and Snellen visual acuity. Diagnosis of anisometropic amblyopia was assigned based on the following: (1) an interocular refractive difference of hyperopia ≥1.00 diopter, astigmatism ≥1.00 diopter, or myopia ≥2.50 diopter; or (2) a history of anisometropia but no history of strabismus or strabismus surgery. Diagnosis of strabismus was made based on strabismus or strabismus surgery, and no anisometropia (as defined above). The clinical data of the subjects with amblyopia and the normal vision subjects are summarized in Table 1. Written informed consent was obtained from the participants’ parents. This study was approved by the Ethics Committee of Sun Yat-sen University in the People’s Republic of China.

**Table 1:** Subjects information.

			**Near VA (logMAR)**		**Near VA (logMAR)**		**Refraction**	
**Subject group**	**Number**	**Age (year)**	**N/F**	**A**	**N/F**	**A**	**N/F**	**A**
Normal control	16	8.50 (1.25)	0.07 (0.20)	—	−0.006 (0.06)	—	0.74 (3.21)	—
Strabismic Amblyopia	17	7.02 (2.30)	0.03 (0.09)	0.5 (0.5)	0.05 (0.08)	0.5 (0.5)	1.02 (0.86)	0.76 (1.25)
Anisometropic Amblyopia	20	8.02 (1.56)	0.02 (0.08)	0.4 (0.2)	0.01 (0.1)	0.4 (0.3)	0.56 (1.11)	0.78 (3.81)

Note: Standard deviations of the means reported in parentheses. VA: visual acuity (best corrected). N: Normal Eye; F: Fellow Eye; A: Amblyopic Eye.

### Visual stimuli and task design

A phase-reversing checkerboard pattern served as the visual stimulus in all experiments. The checkerboard was presented at 15, 30, and 60 s (second of arc) check size corresponding to the visual angle of the fundamental frequency. The contrast was 96%, and the frequency of checkerboard reversal was 2 Hz.

**Figure 1:**
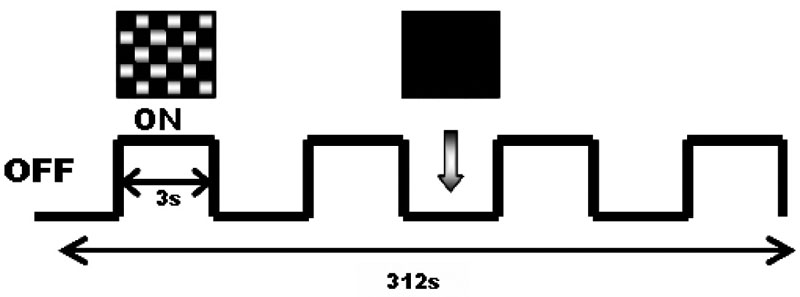
Diagram of blood oxygen level–dependent functional magnetic resonance imaging (BOLD-fMRI) stimulus modality. During the “on” block, a phase-reversing checkerboard pattern was presented, and the “off” block consisted of a black screen with a fixation point.

The experimental task was a short block design, consisting of “on” and “off” periods. The sequence of blocks is shown in Figure 1. During the “on” block, a phase-reversing checkerboard pattern was presented, and the “off” block consisted of a black screen with a fixation point. The total duration of the stimulus presentation was 312 s (each block lasts for 3 s). The block design was presented to each subject twice and averaged.

### Pattern-reversal visual evoked potential data acquisition

PR-VEP recordings were performed with a UTAS-E3000 PR-VEP unit (UTAS-E3000 LKC-Technologies, Gaithersburg, MD). Preparation of the subjects for VEP followed the International Society for Clinical Electrophysiology of Vision guidelines, using Oz as active, Cz as reference, and Pz as ground. The tested eyes were optically corrected, and the binocular viewing condition was adopted. The data acquisition and analysis were implemented with a connected computer. Check-reversal amplitude was the difference between the first major positive peak near 100 ms (P_100_) and the preceding negative peak. The P_100_ amplitude was recorded.

### Blood oxygen level–dependent functional magnetic resonance imaging data acquisition

All MRI scans were performed on a Magneten Siemens Vision 3-T scanner (Siemens AG, Erlangen, Germany). A high-resolution anatomic sagittal T1-weighted three-dimensional magnetization-prepared rapid gradient-echo sequence was obtained for each subject with scanning parameters. The repetition time was 2,000 ms, the echo time was 50 ms, the flip angle was 90°, the matrix was 512×512, the field of view was 24×24 cm^2^, the slice thickness was 1.2 mm, and 124 slices in total were obtained and analyzed. T2*-weighted functional MR images were acquired using gradient-echo echo-planar imaging sequences sensitive to BOLD contrast. The scanned images were reconstructed offline.

During the MR scanning, the subjects with their best-corrected vision were placed in a supine position in the magnet and were instructed to fixate on the center crosshair binocularly at all times. The stimuli were visually projected on a translucent screen at the end of the scanner table using a data projector outside the magnet. The stimuli were shown to the subjects via a mirror positioned above the head coil. A laptop outside the scanner room was connected and exported the data. An MR scan run for each viewing condition (three different check sizes, 15, 30, and 60 s) lasted 312 s, divided into 104 three-second periods. The scanning of the initial 12 s was discarded to eliminate magnetization instability.

Before analyzing the brain images further, we confirmed with a visual inspection that all data sets were uncontaminated with major head motion artifacts. To make sure that the diagnostic subgroups did not differ with respect to amount of head motion, we used the statistical parametric mapping SPM2 software package (SPM2, Wellcome Department of Cognitive Neurology, London, UK) [43] to measure the amount of head motion that occurred during the duration of the scan session. This standard algorithm minimizes the sum of the squared discrepancies between two scans. There was no difference among the three subject groups in measured head motion. To quantify the head motion in each experimental scan, we used the motion-correction algorithm in SPM. For each subject, we extracted the average motion in all three directions as the vector magnitude of translational motion. This value ranged from 0.3 mm to 2.0 mm.

### Image processing and data analysis

We analyzed the data in MATLAB 6.5 (The Mathworks Inc., Natick, MA) using the statistical parametric mapping software SPM2 [43]. We used the MNI Space utility on the MATLAB 6.5 platform to extract the anatomic location of the activation region and export the number of voxels and *Z* values of the activation cluster. The x-, y-, and z-coordinates of the primary maximum in the cluster were labeled as well.

### Region of interest based on analysis of functional imaging data

**Figure 2:**
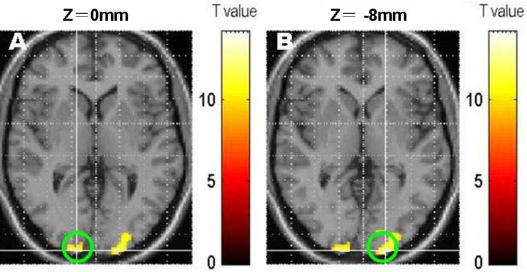
Drawing of the regions of interest. **A**: Homonymous cortex of Brodmann areas (BA) 17; **B**: contralateral cortex of BA 17; the green circle labeled the regions of interest (ROIs).

We used the software program MRIcro to define a three-dimensional calcarine region of interest (ROI). If the amblyopic eye was not the same, for convenience in comparison, we used the software program MRIcro to left-right reverse the brain map. To appropriately compensate for inter-subject anatomic variability, we defined subject-specific ROIs based on anatomic markers to identify functionally equivalent regions across subjects. ROIs were chosen in the gray matter area along the calcarine sulci, which was defined using T1-weighted images obtained on the same slice locations as the echo planar imaging images. Although we did not attempt functional demarcation among the visual areas, we considered that the calcarine ROI most likely included some higher visual areas, such as V2, as well as the primary visual cortex. Because we found a significant difference between the contralateral hemisphere and the ipsilateral hemisphere to the amblyopic eye in this study, we compared the two hemispheres. The calcarine ROI visual areas of BA 17 in the two hemispheres, shown in Table 2 and Figure 2, were drawn for further analysis.

**Table 2:** Z values and localizations of Region of interests.

				**Talairach coordinates**			
**Hemisphere**	**Brain location**	**BA Regain**	**NV**	**x**	**y**	**z**	**Z value**
Homonymy	Cuneus, Lingual Gyrus	17	315	16	−102	0	6.49
Opposite	Lingual Gyrus	17	530	−16	−102	−8	6.06

NV:number of voxels; Z values: peak value of the activation in the of cluster. x, y, z: Talairach coordinates of activation peaks of the cluster.

To analyze the calcarine ROI-based functional imaging data, first applying the calcarine ROI to each subject separately, we extracted the intensity of each voxel within the calcarine ROI. The parameter of the mean MR signal percent change per voxel within the calcarine ROI characterized the mean level of activation. The voxel with the maximum activation and 24 neighboring voxels on the same slice were chosen as a region with maximal response. From the maximal activation region, we computed the mean percent change in the MR signal between the baseline epoch and activation epochs for each patient using the following formula: mean MR signal percent change=(Mean activation intensity of activated states - mean activation intensity of basal states/mean activation intensity of basal states) ×100%.

### Statistical analysis

The group averages in the results were subjected to a general linear model univariate SPM2 procedure performed on the parameter of the mean MR signal percent change, using population (normal vision group, anisometropic, and patients with strabismic amblyopia) and stimuli (three spatial frequencies) as factors. This analysis permitted the assessment of the level of activation differences among groups as well as the stimuli. We analyzed the correlation between the cortical activation in fMRI and the P_100_ amplitude in VEP using the SPSS 12.0 software package (SPSS Inc., Chicago, IL). A value of p<0.05 was considered statistically significant.

## Results

### Blood oxygen level–dependent response location in patients with anisometropic and strabismic amblyopia

After the SPM2 analysis, we overlaid the significant differences in the activation maps of the subjects’ BOLD-fMRI results onto the average anatomic images. Only those voxels that surpassed a threshold of p=0.05 were considered activated areas overlaid by the visual task. In the stimulation-rest comparisons, significant voxel clusters were located in Brodmann’s areas (BA) 17 and 18, BA 19, the ventroposterior area, and the occipitotemporal junction region in BA 37 bilaterally. We also found some activation in the parietal lobe. The cortical activation in the subjects with amblyopia, due to either anisometropia or strabismus, showed a reduced response in strength and area compared to the normal control eyes.

**Figure 3:**
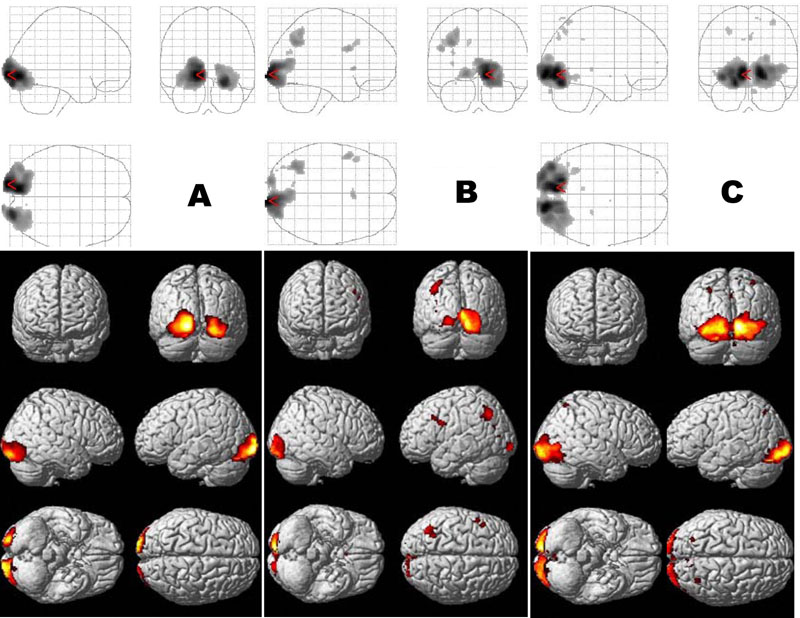
Localization of statistically significant reductions in the visual cortex of the subjects with anisometropic amblyopia compared with normal vision subjects. **A**: Stimulus of 60 s check size; **B**: stimulus of 30 s check size; **C**: stimulus of 15 s check size.

**Figure 4:**
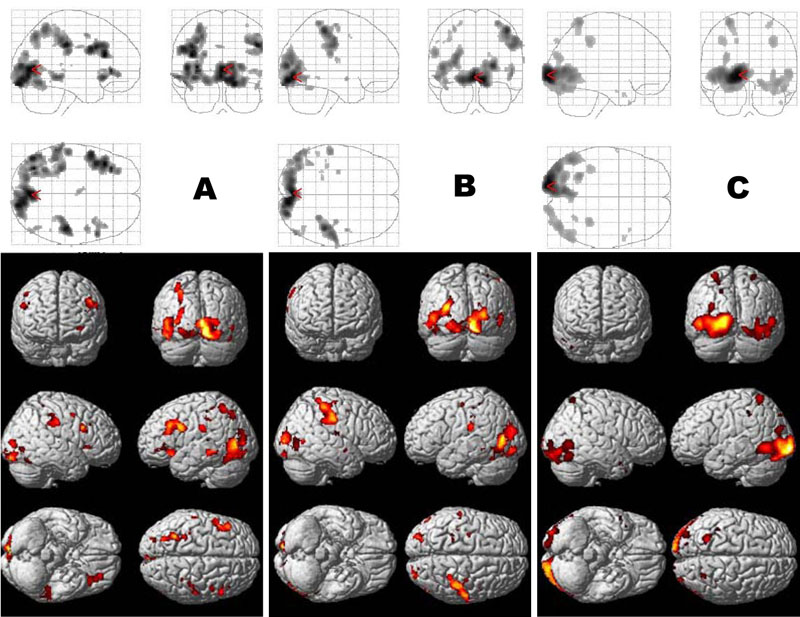
Localization of statistically significant reductions in the visual cortex of the subjects with strabismic amblyopia compared with normal subjects. **A**: Stimulus of 60 s check size; **B**: stimulus of 30 s check size; **C**: stimulus of 15 s check size.

The cortical activation evoked by the anisometropic amblyopic eye was significantly reduced in bilateral BA 17 with a small significant change in BA 18 and 19. The reduced areas were similar at low- and middle-spatial-frequency stimulation (check size: 60 s, 30 s), whereas a significant reduction was found at high spatial frequency (check size: 15 s) in bilateral BA 17 (Figure 3). However, for the strabismic amblyopic eye, the cortical activation was significantly reduced in bilateral BA 18 and 19, with a small significant change in BA 17, and even in the temporal lobe and the parietal lobe. There were no significantly reduced areas at low- and middle-spatial-frequency stimulation (check size: 60 s, 30 s), whereas a significant reduction was found at high spatial frequency (check size: 15 s) in bilateral BA 18 and 19 (Figure 4).

**Figure 5:**
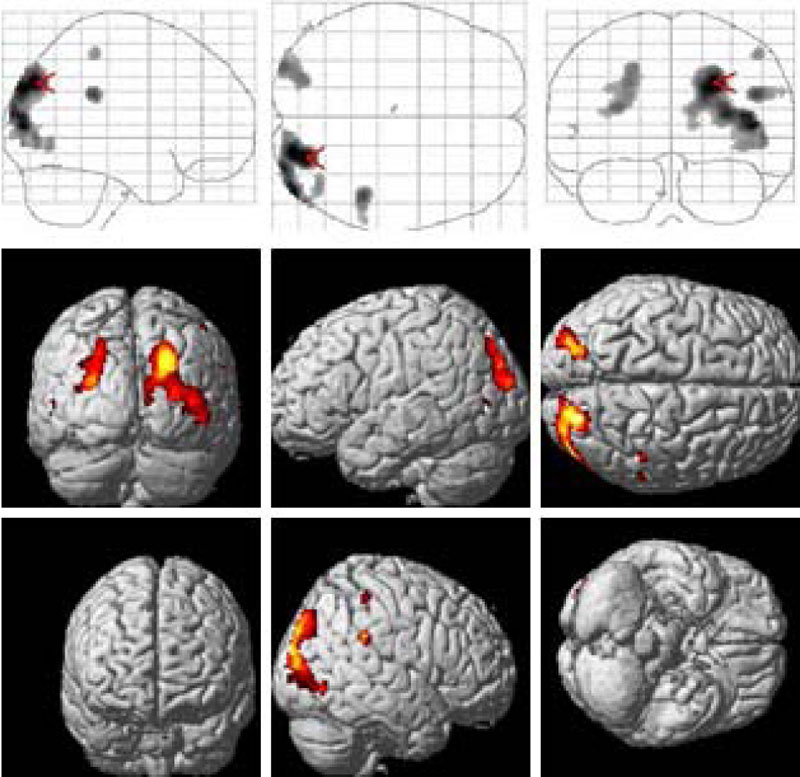
Difference in the two types of amblyopia showing the localization of statistically significant reductions in the visual cortex of the subjects with strabismic amblyopia compared with that of the subjects with anisometropic amblyopia. The cortical response evoked in patients with strabismic amblyopia showed a more significant reduction than that in the patients with anisometropic amblyopia group in extrastriate areas, including Brodmann areas (BA) 18 and 19, the temporal lobe, and the parietal lobe at all the stimulus of 60 s, 30 s, and 15 s check size.

When the two types of amblyopia were compared, the cortical response evoked by the strabismic amblyopia group showed a more significant reduction than that of the anisometropic amblyopia group in extrastriate areas, including BA 18 and 19, as well as the temporal and parietal lobes, including BA 37, BA 21, BA 7, BA 20, BA 39, etc. (Figure 5) at all the stimulus of the 60 s, 30 s, and 15 s check size.

### Region of interest–based analysis of functional imaging data to compare the differences in spatial frequency–dependent magnetic resonance imaging cortical activation

**Figure 6:**
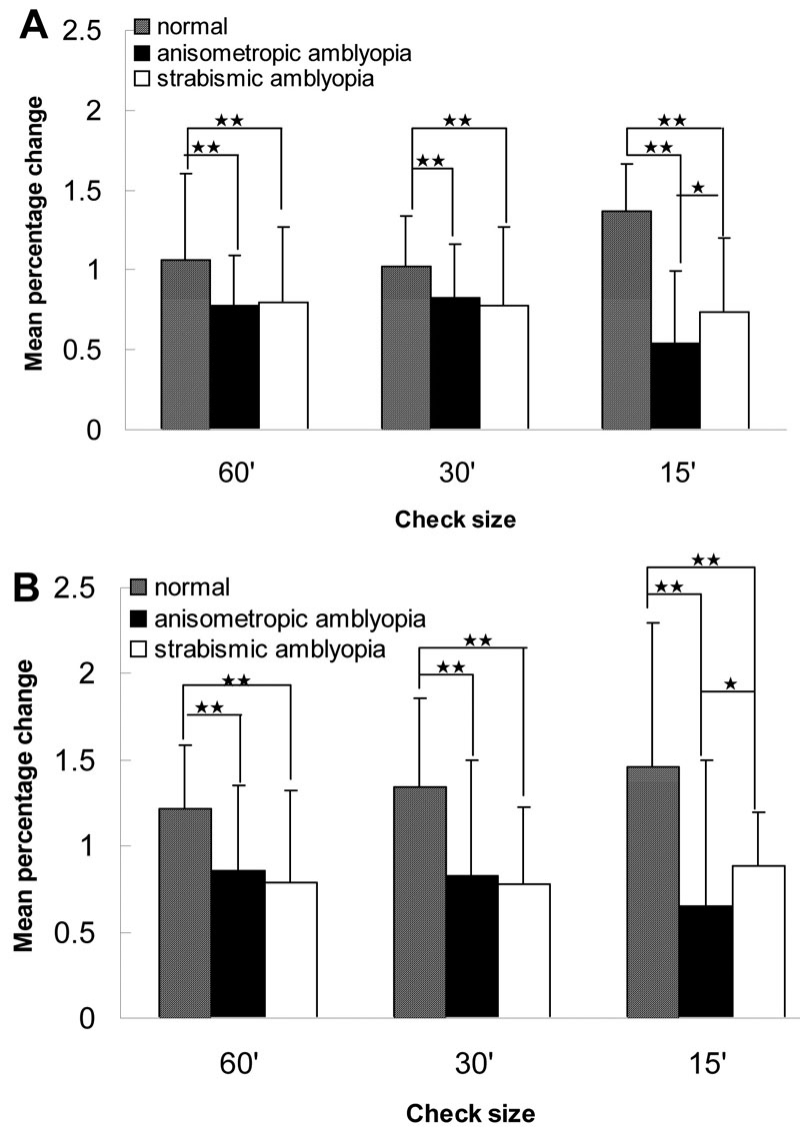
Difference in spatial frequency–dependent magnetic resonance imaging cortical activation. The percent change in the magnetic resonance (MR) signal is plotted as a function of the spatial frequency of the checkerboard pattern. Compared to the normal vision control group, the mean percentage change decreased significantly in the amblyopia groups across a range of spatial frequencies. There was no significant difference between the strabismic group and the anisometropic group at 60 s and 30 s checkerboard patterns, while the anisometropia reduced the cortical response to the 15 s checkerboard pattern (high-spatial-frequency) stimulus in the bilateral Brodmann areas (BA) 17. anisometropic amblyopia: 20 subjects; strabismic amblyopia: 17 subjects; normal vision volunteers: 16 subjects. *means p<0.05, **means p<0.01.

We used the grand linear model procedure to compare the mean MR signal percentage change within the calcarine ROIs of BA 17 across the groups with three spatial frequencies of the checkerboard pattern. The results are shown in Figure 6.

The plotted percentage changes in the MR signal with the spatial frequency of the checkerboard pattern are shown in Figure 6. In BA 17, contralateral to amblyopic eyes when compared, compared to the normal vision control group (the right hemisphere), the mean percentage change decreased significantly in the strabismic amblyopia group (60 s: p=0.000; 30 s: p=0.000; 15 s: p=0.000) and the anisometropic amblyopia group (60 s: p=0.000; 30 s: p=0.001; 15 s: p=0.001) across a range of spatial frequencies. There was no significant difference between the strabismic group and the anisometropic group at the 60 s (p=0.566) or 30 s (p=0.067) checkerboard patterns. However, anisometropia reduced the cortical response at the 15 s checkerboard pattern (high-spatial-frequency) stimulus in BA 17 compared to the strabismic group (p=0.017; Figure 6A).

There was a similar change in BA 17 ipsilateral to the amblyopic eyes. Compared to the normal vision control group (the left hemisphere), the mean percentage change decreased significantly in the strabismic amblyopia group (60 s: p=0.000; 30s: p=0.005; 15 s: p=0.000) and the anisometropic amblyopia group (60 s: p=0.000; 30 s: p=0.000; 15 s: p=0.001) across a range of spatial frequencies. Although at 60 s or 30 s checkerboard patterns, there was no significant difference between the strabismic group and the anisometropic group (p=0.321 and p=0.085); anisometropia reduced the cortical response at the 15 s checkerboard pattern (high-spatial-frequency) stimulus in BA 17 (p=0.031; Figure 6B).

### Difference in spatial frequency–dependent VEP cortical response

**Figure 7:**
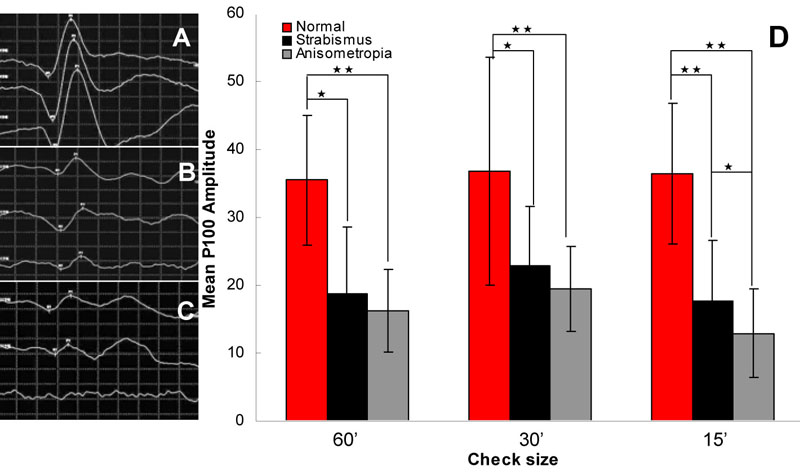
Oscillogram of pattern-reversal visual evoked potential (PR-VEP) and P_100_ amplitude in PR-VEP. **A**: Normal control group (n=16); **B**: strabismic amblyopia group (n=17); **C**: anisometropic amblyopia group (n=20). **D**: P_100_ amplitude in PR-VEP. We can see, using high spatial frequency, the reduced amplitudes of anisometropic amblyopia group were more significant than those of the other groups. *means p<0.05, **means p<0.01.

Compared with the normal vision control group, both amblyopic groups showed significant reduction in the three spatial frequency stimuli. The anisometropic amblyopia group showed significant reduction only in the 15 s checkerboard pattern (p=0.029) compared to the strabismic amblyopia group. The strabismic amblyopia group showed no statistically significant difference between the three spatial frequency stimuli (p=0.513, p=0.499, p=0.293). The anisometropic amblyopia group, on the other hand, showed a significant reduction in the 15 s checkerboard pattern (high-spatial-frequency) stimulus compared with the low and middle spatial frequencies (p=0.034, p=0.001). The difference between the low and middle spatial frequencies was not significant (p=0.183). (Table 3, Figure 7).

**Table 3:** P100 amplitude of pattern-reversal visual evoked potential (PR-VEP).

	**Check size**		
**Group**	**60’**	**30’**	**15’**
Normal control	35.45 (9.50)	36.83(16.77)	36.43(10.33)
Strabismic amblyopia	18.83 (9.68)	22.78 (8.85)	17.66 (8.88)
Anisometropic amblyopia	16.29 (6.11)	19.42 (6.25)	12.92 (6.55)

Standard deviations of the means reported in parentheses.

### Correlation analysis between the activation of the blood oxygen level–dependent functional magnetic resonance imaging and the P_100_ amplitude in visual evoked potential

**Figure 8:**
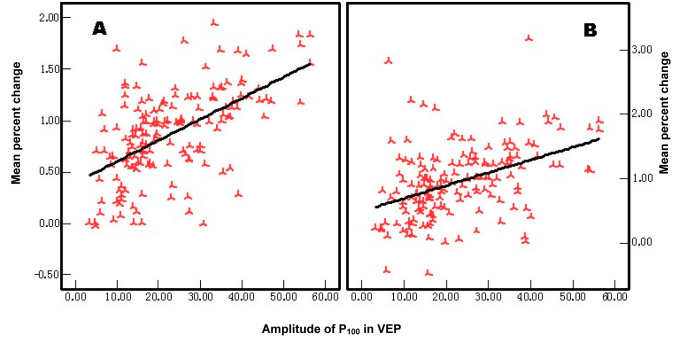
Correlation between the P_100_ amplitude in pattern-reversal visual evoked potential (PR-VEP) and the activation of cortical neurons. **A**: Opposite hemicerebrum; **B**: Homonym hemicerebrum. The bilateral cortical mean percent change of the blood oxygen level–dependent functional magnetic resonance imaging (BOLD-fMRI) was significantly correlated with the P_100_ amplitude in VEP, in the contralateral and the ipsilateral Brodmann area (BA).

Because we found that there was a significant difference between the contralateral hemisphere and the ipsilateral hemisphere in the amblyopic eye in the trial, we compared the two hemispheres to one another. The unilateral cortical mean percent change of the BOLD-fMRI was significantly correlated with the P_100_ amplitude in VEP in the contralateral (r=0.421, p=0.000) and ipsilateral BA 17s (r=0.562, p=0.000; Figure 8).

## Discussion

The results of this study demonstrate a successful combination of the spatial frequency–dependent BOLD-fMRI and PR-VEP cortical response to the same pattern of stimuli in the same juvenile subjects of amblyopia and the normal vision group. The effect of spatial frequencies and the localization of the cortical deficit were discussed in anisometropia and strabismus amblyopia, and the results of this study clarify the etiopathogenesis of these forms of amblyopia. Earlier studies chose adult participants as study subjects. They showed good compliance, and the cortex agreed with the Talairach atlas in the SPM2 software, but the age span was about 30 years, so that there was low comparability. For the current study, we choose seven- to 12-year-old juvenile subjects as study subjects; since they were almost at the same age level, the comparability was better. They also exhibited good compliance, and their brain development suited SPM2 software analysis as well as that of adults. In addition, in this study all the subjects were tested under binocular viewing conditions, which allowed better comparison and analysis of the amblyopia binocular influence in the visual cortex. Previous studies showed that psychophysical and functional imaging techniques, such as fMRI, positron emission tomography [25,44], single photon emission computed tomography [27], and magnetoencephalography [26], have been widely used to observe the site of cortical dysfunction in patients with strabismic and anisometropic amblyopia. Yet this research has come to no consistent conclusion. The earlier studies using monkeys and kittens [13,16,18] tended to place the principal abnormality at the level of the primary visual cortex (BA 17), while others showed a reduced response in the extrastriate cortex [25,45–47]. This study combined medical imaging and electrophysiological techniques to localize the cortical deficit in amblyopia. More subtle localization characteristics were brought to light. First, BA 17 and other extrastriate areas showed reduced areas and levels of activation in amblyopia (due to either anisometropia or strabismus) at all spatial frequencies. This indicated that BA 17 is not the only area affected in the amblyopic visual cortex. Second, using spatial frequency–dependent observation to detect subtle activation differences, the two types of amblyopia showed a reduced fMRI response over the entire frequency range, but they still exhibited differences in the comparison across the spatial frequencies. A reduced fMRI response was observed over the entire frequency range, particularly at high spatial frequency, in human patients with anisometropic amblyopia, and the reduced area mainly resided in the striate visual cortex (the primary visual cortex or BA 17). Compared with patients with anisometropic amblyopia, patients with strabismic amblyopia exhibited many different characteristics. In bilateral BA 18 and 19, we found a more significant cortical impairment than that in patients with anisometropic amblyopia. In the spatial frequency–dependent observation, patients with strabismic amblyopia showed a significant reduction at high spatial frequency only in the extrastriate cortex. At high spatial frequency, patients with anisometropic amblyopia showed significantly reduced activation compared with other spatial frequencies in the striate cortex as well as the extrastriate cortex, whereas patients with strabismic amblyopia showed a significant reduction at high spatial frequency only in the extrastriate cortex.

Amblyopia is characterized as the loss of spatial resolution. The dysfunction of BA 17 may be sufficient to explain this characteristic. However, this loss in amblyopia does not adequately represent the full extent of the visual dysfunction. There are numerous examples of reduced performance by an amblyopic visual system. These performances include position [48–51], orientation [52], shape [53], second-order detection [54,55], and motion adaptation [56]. The result of this study is consistent with neurophysiological findings in recent years that the primary visual cortex is the principal site of the visual loss in amblyopia, yet there are definitely additional deficits at higher levels of the visual pathways. Strabismic amblyopia in humans is composed of two different and possibly unrelated deficits: reduced contrast sensitivity [46,57] and increased positional uncertainty [48,58]. The neurophysiological basis of these deficits is still unclear. Some reports have suggested that the reduced brain activation is confined to the calcarine cortex, while others have shown a reduced response in the extrastriate cortex [25,45]. Using contrast sensitivity to detect patients with strabismic amblyopia, such sensitivity was found to be depressed for only a limited band of high spatial frequencies, while in patients with anisometropic amblyopia, depression of the contrast sensitivity function was found over the entire frequency range [59]. Some previous fMRI findings suggested that only patients with anisometropic amblyopia show a reduced fMRI response in the high spatial frequency range, while no significant differences between the amblyopic and normal eye were observed in patients with strabismic amblyopia. In the current study, we found the level of this reduction was similar in the striate and extrastriate cortex; moreover, the activation was reduced more significantly in the extrastriate cortex of patients with strabismic amblyopia than in that of patients with anisometropic amblyopia. This finding seemed to support the notion that strabismic amblyopia may result from the breakdown of binocular interaction thought to be correlated with the extrastriate cortex [60], while anisometropic amblyopia may be a result of selective undersampling of a visual image at high spatial frequencies.

In the present study, we introduced a method that combines information from VEP and fMRI measurements performed separately but on the same subject and using the same stimuli. In this way, the first attempt to detect visual deficits in anisometropic and strabismic amblyopia was conducted. A combination of the methods could noninvasively localize synaptic activity changes in space and time, and thus further enlarge the range and scope of functional imaging studies in ophthalmology and neuroscience.
